# Bile acid metabolism and hepatocellular carcinoma: mechanisms of drug resistance and intervention strategies

**DOI:** 10.1093/pcmedi/pbaf020

**Published:** 2025-08-20

**Authors:** Yan Lu, Xiaochen Feng, Zhijie Wang, Minghao Zou, Zheqi Xu, Qianjia Liu, Wenjin Chen, Jin Ding, Hui Liu

**Affiliations:** School of Health Science and Engineering, University of Shanghai for Science and Technology, Shanghai 200093, China; The Third Department of Hepatic Surgery, Eastern Hepatobiliary Surgery Hospital, Shanghai 200438, China; Clinical Cancer Institute Center for Translational Medicine, Naval Medical University, Shanghai 200433, China; The Third Department of Hepatic Surgery, Eastern Hepatobiliary Surgery Hospital, Shanghai 200438, China; The Third Department of Hepatic Surgery, Eastern Hepatobiliary Surgery Hospital, Shanghai 200438, China; The Third Department of Hepatic Surgery, Eastern Hepatobiliary Surgery Hospital, Shanghai 200438, China; Department of Urology, Xinhua Hospital, School of Medicine, Shanghai Jiaotong University, Shanghai 200092, China; Clinical Cancer Institute Center for Translational Medicine, Naval Medical University, Shanghai 200433, China; School of Health Science and Engineering, University of Shanghai for Science and Technology, Shanghai 200093, China; The Third Department of Hepatic Surgery, Eastern Hepatobiliary Surgery Hospital, Shanghai 200438, China

**Keywords:** bile acid, hepatocellular carcinoma, drug resistance, immunotherapy, targeted therapy, tumor metabolism

## Abstract

Hepatocellular carcinoma (HCC) is the predominant malignant liver tumor, characterized by high morbidity, mortality, and rapid progression, and it ranks among the leading causes of cancer-related fatalities worldwide. Its treatment is facing the severe challenge of resistance to targeted drugs and immunotherapy. Bile acids (BAs) are products of cholesterol metabolism, that not only regulate lipid digestion and absorption, but also influence the development of HCC by modulating inflammation and metabolism. Dysregulation of BA metabolism is closely linked to resistance against targeted therapies and immunotherapies. BAs reduce the efficacy of targeted drugs by influencing enzymes involved in drug metabolism and drug efflux transporters, moreover, BAs also lead to immunotherapeutic resistance by regulating the formation of the immunosuppressive tumor microenvironment. Therefore, regulating BA metabolism has the potential to overcome drug resistance of targeted therapy and immunotherapy, which could be a promising treatment strategy. This review not only summarizes the roles of BA metabolism in HCC development and drug resistance, but also further explores the rationality and necessity of targeting BAs to enhance the survival of HCC patients.

## Graphical Abstract

**Graphical Abstract:**
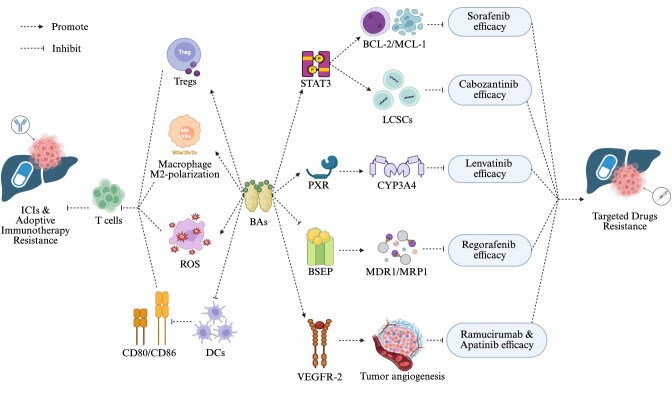
The mechanism of bile acids-mediated drug resistance in hepatocellular carcinoma.

## Introduction

Liver cancer is the eighth most prevalent cancer and the third leading cause of carcinoma-associated morality globally [1]. Hepatocellular carcinoma (HCC), which is the predominant histological sub-type, constitutes ∼90% of all primary liver cancer cases [2]. The risk factors for HCC primarily include alcohol consumption, viral infections (e.g. hepatitis B and C viruses), metabolic dysfunction-associated steatohepatitis (MASLD), autoimmune hepatitis cholestatic liver disorders, etc [3]. Drug therapy, primarily including targeted drugs and immunotherapy, is one of the most important means of treating HCC, but they all face serious drug resistance problems [4]. These systemic drugs can only prolong survival by a few months, as tumors are always developing [5]. Therefore, studying drug resistance mechanisms and developing new treatment strategies are important research directions in HCC treatment.

Tumor metabolism has appeared as a focal point of contemporary oncology research. Tumor cells achieve their growth needs and drug therapy sensitivity through metabolic reprogramming [6]. Bile acids (BAs), as pivotal metabolic regulators in hepatic physiology, have modulated therapeutic efficacy in HCC by changing their metabolic circuitry. BAs, which are steroid derivatives that are synthesized in the liver, facilitate lipid digestion/absorption in the intestine and regulate metabolism via nuclear receptors (NRs). Gut microbiota convert primary BAs (PBAs) into secondary forms, thereby improving their amphipathic properties for efficient micelle formation and systemic signaling [7–9]. BA metabolism imbalance affects HCC development and the treatment efficacy of related HCC drugs [10, 11]. This review aims to give a complete outline of the mechanisms of BA metabolism in HCC and provide an in-depth analysis of how BAs can cause resistance to targeted and immunotherapies in HCC, while proposing combination therapy strategies and assessing their potential to address drug resistance.

## BA metabolism and HCC

BAs are the main metabolic end products of cholesterol, comprising PBAs and secondary bile acids (SBAs), which have basic physiological functions, including promoting fat digestion and absorption, regulating cholesterol metabolism, and maintaining intestinal flora balance [12]. PBAs are kept in the gallbladder for a short period of time, released into the intestine in response to dietary fats, and later returned to the liver through the portal vein, which involves various transport proteins to finalize the enterohepatic cycle. The unabsorbed BAs travel to the colon and are transformed into SBAs by the gut microbiota [13]. Altered BA composition and hydrophobicity, as well as disturbances in metabolism and signaling, are part of the pathogenesis of MASLD, cirrhosis, and HCC [10, 14]. BA homeostasis imbalance promotes hepatocyte death and malignant transformation by activating the inflammasome, thereby inducing HCC occurrence [15].

### Abnormal BA receptor signaling

BAs are important signaling molecules that can affect substance metabolism, inflammatory responses, etc. by activating corresponding receptors [16, 17]. Relevant studies have shown that hepatic farnesoid X receptor (FXR) expression is downregulated in HCC patient samples and further contributes to a worse prognosis in HCC [18, 19]. In 2007, a study indicated that deletion of FXR expression elevates the level of BAs and further promotes the development and progression of HCC [20]. In parallel, a study by Kim *et al*. [17] demonstrated that cytotoxic BAs accumulate in the liver of FXR−/− mice, thereby inducing the inflammatory signal Interleukin (IL)-1β, which indirectly leads to the expression of the cell proliferation gene β-catenin and its target gene, myelocytomatosis oncogene (c-Myc), ultimately causing the development of HCC. In addition, accumulated bile salts, such as glycochenodeoxycholic acid (GCDC), promote tumor cell survival and confer resistance to chemotherapy through the extracellular signal-regulated kinase/myeloid cell leukemia-1 (ERK/MCL-1) signaling pathway [21]. As reported by Anakk *et al*. [22], double knockout of FXR and small heterodimer partner (SHP) increases BA levels, thereby activating the YAP signaling pathway and leading to the occurrence of HCC. FXR also alters the expression levels of tumor suppressors such as suppressor of cytokine signaling 3 (SOCS3), N-Myc downstream-regulated gene 2, and microRNA-122, which further affect the proliferation of HCC cells [23–25].

The G protein-coupled bile acid receptor (TGR5) regulates the metabolic homeostasis of BAs [26, 27]. The changes of TGR5 expression are complex in the livers of HCC patient. Previous investigations have documented elevated TGR5 expression levels in neoplastic tissues compared to their normal counterparts, and high TGR5 expression is associated with a favorable prognosis [28]. Another study found that TGR5 promoter was abnormally hypermethylated in the serum of HCC patients, implying its potential as a new diagnostic biomarker [26]. While the direct mechanism by which altered TGR5 expression causes HCC is still unclear, TGR5 deficiency makes mice more vulnerable to infection, inflammation, and cholestatic liver injury [29]. In 2011, Wang *et al*. [30] reported that compared with wild type (WT) mice, in TGR5−/− mice, macrophages, primary Kupffer cells, and livers exhibited increased mRNA levels of pro-inflammatory nuclear factor kappa-B (NF-κB) target genes. In addition NF-κB can activate cytokine IL-6 by regulating signal transducer and activator of transcription 3 (STAT3), which ultimately boosts HCC development [31].

Vitamin D3 receptor (VDR) is another NR for BAs [32]. The only BA that can activate the VDR is lithocholic acid (LCA) and its major metabolites 3-keto-LCA, glyco-LCA, and 6-keto-LCA [33]. As reported by Li *et al*. [34], transcriptional regulation of the VDR gene is directly mediated by the tumor suppressor Krüppel-like factor 4 (KLF4) via promoter binding, and deletion of KLF4 expression inhibits VDR expression and activation and causes HCC pathogenesis. Pregnane X receptor (PXR) functions as a master xenobiotic sensor that orchestrates detoxification through transcriptional activation of phase I/II metabolic enzymes [cytochrome P450 3A4 (CYP3A4) and uridine diphosphate glucuronosy ltransferase 1A1 (UGT1A1)] and efflux transporters [ATP-binding cassette sub-family B member 1 (ABCB1) and multi-drug resistance protein 1 (MDR1)], thereby enhancing the metabolism and biliary excretion of toxic compounds [35]. PXR is expressed in liver and is a low-affinity NR for a subset of BAs, activated primarily by LCA and its 3-keto metabolite [32, 36]. Activation of PXR increases HCC resistance to multiple chemotherapeutic agents. Specifically, in HCC cells, upregulated LCA activates the expression of PXR, which in turn stimulates the expression of its downstream genes (e.g. CYP3A4 and MDR1) to interfere with the metabolism and clearance of toxic compounds (e.g. anti-tumor drugs), ultimately leading to drug resistance in HCC [37–39].

**Figure 1.**
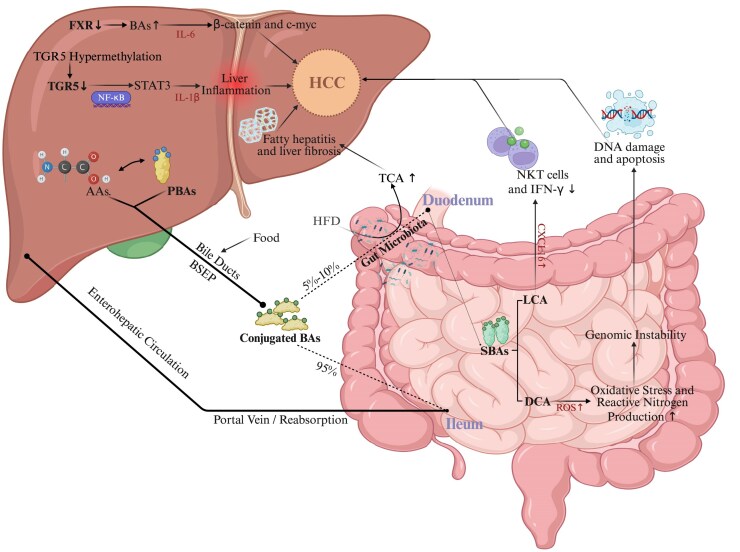
Mechanisms of BA metabolism in the development of HCC. AAs, amino acids; BSEP, bile salt export pump; DCA, deoxycholic acid; HFD, high-fat diet; IFN-γ, interferon-γ; NKT cells, natural killer T cells; ROS, reactive oxygen species; TCA, taurocholic acid.

In summary, BA receptors play an important role in the development of HCC by regulating processes such as metabolism, inflammation, and immune response in the liver (Fig. 1).

### BA-mediated dysbiosis of intestinal flora

The gut microbiota affects HCC progression by regulating the transformation of BAs. PBAs are synthesized in hepatocytes, conjugated with amino acids (AA), and afterwards carried into the bile ducts through bile salt export pumps (BSEPs) [40, 41]. After food intake, BAs are released into the duodenum. Approximately 95% of conjugated BAs undergo re-absorption in the ileum, followed by circulation to the liver through portal blood flow, with this entire process collectively designated as the enterohepatic cycle [42]. Overall, 5% to 10% of unresorbed BAs can serve as a substrate for gut microbiota metabolism, and can be biotransformed into SBAs by enzymatic modifications that shape the biological activity of BAs [43, 44]. The physicochemical properties of some BAs are changed by intestinal microbiota. Conversely, BAs also impact the gut microbiota's configuration and role [45]. A related study concluded that the gut microbiota could boost HCC's pathogenesis via the enterohepatic axis, and probiotics could inhibit the growth of HCC and tumor angiogenesis [46].

The gut microbiota can convert primary bile salts into SBAs through depolymerization by bile salt hydrolase (BSH) and 7α-dehydroxylation mediated by 7α-dehydroxylase [8, 46]. The research indicated that gut microbial dysbiosis frequently occurs in cirrhotic-HCC patients, manifesting as a significant proliferation of Enterobacter, Enterococcus, and Clostridium genera. These BSH-rich, aerobic bacteria drive pro-inflammatory responses and contribute to increased SBA synthesis [11, 47]. High concentration SBAs such as deoxycholic acid (DCA) and LCA exert cytotoxic effects through oxidative stress, DNA damage, and inflammatory pathways, ultimately leading to carcinogenesis [48]. It has been reported that DCA leads to genomic instability, and the mechanism involves reactive oxygen species (ROS)-induced oxidative stress and reactive nitrogen production, which generate DNA damage and apoptosis [49, 50]. Long-term DNA damage leads to an increase in genetic mutations, which ultimately promotes the development of tumor cells [51]. Meanwhile, Yoshimoto *et al*. [52] demonstrated that DCA induces a senescence-associated secretory phenotype in hepatic stellate cells, which promotes the development of obesity-associated HCC. In addition, the report summarized that increased LCA inhibits the expression of C-X-C motif chemokine ligand 16 (CXCL16), a separate ligand of CXCR6, which suppresses hepatic CSCR6+ natural killer T (NKT) cell aggregation and interferon γ (IFN-γ) production, and promotes HCC development [53]. MAF bZIP transcription factor G (MAFG) as the key regulatory factor in BAs has an influence in inflammation and liver injury [54]. Some results showed that the up-regulation of MAFG was related to poor prognosis in HCC, and LCA induces MAFG expression by activating enhancer elements of the MAFG promoter, NF-κB and E-box, thus promoting the development of HCC [55].

High-fat diet (HFD) refers to a high-fat dietary regimen inducing obesity/metabolic disorders. HFD affects the metabolism of related BAs through intestinal bacteria, and also alters the gut barrier permeability, which causes inflammation and further promotes HCC development. The level of gut bacterial metabolite taurocholic acid (TCA) increased in the serum of mice fed by HFD, while the level of 3-indolepropionic acid (IPA) decreased [56]. Of note, increase in TCA or decrease in IPA induces hepatic lipid accumulation, inflammation, cell proliferation, and ultimately leads to HCC [53]. *In vitro* experiments have confirmed that, TCA potentiates the accumulation of triglycerides caused by cholesterol in human LO2 cells, while IPA inhibits the accumulation of triglycerides in nonalcoholic steatohepatitis (NASH)-HCC cells. This further promotes the progression of fatty liver to a more severe stage such as steatohepatitis and fibrosis. Subsequently, the increased proliferation of hepatocytes further leads to HCC development [56].

In conclusion, intestinal microbiota dysfunction transforms PBAs into more hydrophobic SBAs, e.g. DCA and LCA, which lead to DNA damage, cellular senescence, and activation of inflammatory signaling pathways, thereby speeding up HCC growth (Fig. 1).

### Bidirectional role of BA metabolism-related enzymes

BA metabolism-related enzymes play a bidirectional role in mediating HCC progression, not only promoting HCC progression but also inhibiting HCC development.

In the classical pathway of BA synthesis, the enzyme cholesterol 7α-hydroxylase (CYP7A1) catalyzes the conversion of cholesterol into 7α-hydroxycholesterol, which subsequently produces two types of PBA: cholic acid (CA) and chenodeoxycholic acid (CDCA), the ratio of which is controlled by the catalytic intermediate 12α-hydroxylation of sterol 12α-hydroxylase cytochrome P450 family 8B1 (CYP8B1) [57, 58]. The related study showed that CYP8B1 expression is reduced in HCC patients and leads to an increase in the hydrophilic proportion of BA components [59]. In parallel, relevant experiments reported that elevated levels of CDCA in the livers of mice treated with tauro-chendoxycholic acid (TCDCA) and tauro-ursodeoxycholic acid (TUDCA) inhibit the expression of CYP8B1 in the classical pathway and further promote the development of HCC [60]. Another alternative pathway for BA synthesis begins with the hydroxylation of the cholesterol side chain by the mitochondrial cytochrome P450 sterol 27-hydroxylase (CYP27A1) to generate 27-hydroxycholesterol and ultimately CDCA-based BAs [61]. The study concluded that alternative pathways of BA synthesis are upregulated in advanced stages of liver disease, and that CYP27A1 increases the production of the endogenous agonists 25-oxysterol (25-OHC) and 26-oxysterol (26-OHC), which activates the liver X receptor (LXR), promotes the expression of liposynthesis enzymes, and affects the collection of fats in the liver, resulting in NASH and accelerating the development of HCC [62]. In addition, CYP27A1 downregulation leads to an imbalance in cholesterol metabolism, exacerbates liver injury and fibrosis, and ultimately promotes HCC [63]. Therefore, the dysregulation of BA anabolic enzymes leads to hepatic fat accumulation, elevated oxidized cholesterol levels, and inflammatory responses, thereby advancing the occurrence and development of HCC.

The BA metabolism enzyme aldoketo reductase 1D1 (AKR1D1) inhibits the accumulation of CDCA and the proliferation of *Bacteroides ovatus*, thereby slowing down the metabolism of CDCA to isolithocholic acid (iso-LCA) and maintaining the tumor-killing ability of NK cells [64]. Therefore, BAs can regulate HCC immunity through the AKR1D1–*B. ovatus*–iso-LCA–NK cell axis. In addition, the conversion of PBAs into SBAs mediated by gut microbiota can regulate the expression of CXCL16, which in turn regulates the accumulation of C-X-C chemokine receptor type 6+ (CXCR6+) NKT cells in the liver, thereby inducing liver-specific anti-tumor effects and inhibiting HCC progression [65]. The conjugation of BAs and AAs is catalyzed by BA-CoA, amino acid *N*-acyl transferase (BAAT). The study has shown that knocking out BAAT inhibits the synthesis of conjugated BAs in hepatocytes, enhances tumor-specific T cell responses, inhibits tumor growth, and increases tumor sensitivity to immunotherapy [66]. The expression level of cytochrome P450 2E1 (CYP2E1) in tumors is negatively correlated with serum total bile acid (TBA) levels. A decrease in TBA leads to upregulation of CYP2E1, which inhibits HCC cell proliferation, blocks cholic acid (CA)-activated autophagy, and reduces protein kinase B (AKT) phosphorylation through the AKT/mTOR signaling pathway [67]. Therefore, enzymes related to BA metabolism also inhibit HCC progression through multiple pathways, thereby exerting an anti-tumor effect.

## BA metabolism and related genes in HCC-targeted drug resistance

Emerging evidence emphasizes that dysregulated BA metabolism contributes to therapeutic resistance in HCC through multifaceted mechanisms. These include BA-receptor-mediated oncogenic signaling pathway activation (e.g. FXR, PXR), drug-metabolizing enzyme (DME) modulation (e.g. CYP450 family), and altered drug efflux transporter expression [e.g. multi-drug resistance-associated proteins (MRPs)]. Such BA-driven adaptive responses not only compromise the efficacy of targeted treatments, but also promote tumor survival under pharmacological pressure. The following subsections systematically dissect the interplay between BA metabolism and resistance to specific agents, beginning with the first-line tyrosine kinase inhibitors (TKIs) sorafenib and lenvatinib, followed by other targeted drugs (Table 1).

**Table 1.** BA metabolism-driven targeting and immunotherapy resistance mechanisms^a^.

Drug type		Drug name	Main mechanisms of BA-mediated drug resistance in HCC	Ref.
Targeted drugs		Sorafenib	HCC → FXR−/− → Increased levels of BAs → Activate STAT3 → Increased expression of BCL-2 and MCL-1 → Reduced the pro-apoptotic effects of sorafenib → HCC drug resistance	[70, 71]
	Lenvatinib	BAs → PXR activation → Induction of CYP3A4 expression → Influence on the pharmacokinetics of lenvatinib → HCC drug resistance	[80–82]	
	Regorafenib	HCC → FXR−/− → BSEP functional deficiency → Upregulation of MDR1 and MRP1 expression → Regorafenib efflux increased → Intracellular regorafenib concentration reduced → HCC drug resistance	[84–86]	
	Cabozantinib	GCDC → Activation of EMT and STAT3 signaling pathway → Induction of LCSCs → HCC insensitive to cabozantinib	[87, 88]	
	Ramucirumab and Apatinib	BAs → Increased VEGFR-2 expression → Promotion of HCC tumor angiogenesis → Inhibition of ramucirumab and apatinib efficacy → HCC drug resistance	[91–94]	
Immuno-therapy	Immune checkpoint inhibitors	PD-1/PD-L1 inhibitors	① LCA derivative → Promotion of mitoROS generation → Increased FoxP3 expression → Enhanced differentiation of Tregs → Reduced efficacy of PD-1/PD-L1 inhibitors → HCC drug resistance	[99, 100]
			② UDCA → Macrophage polarization to M2 type → Promoting the production of IL-10 and TGF-β → Suppression of effector T cells → Reduced efficacy of PD-1/PD-L1 inhibitors → HCC drug resistance	[101, 102]
		CTLA-4 inhibitors	TCDCA → Inhibition of mitochondrial respiration→ ROS increased →Impairment of T-cells → Reduced efficacy of CTLA-4 inhibitors → HCC drug resistance	[66]
		LAG-3 inhibitors	DCA → Impairment of DCs → Reduced CD80/CD86 expression → Decreased T-cell reactivity → Reduced efficacy of LAG-3 inhibitors → HCC drug resistance	[110–112]
		TIM-3 inhibitors	UDCA → Macrophage polarization to M2 type → Suppression of effector T cells → Reduced efficacy of TIM-3 inhibitors → HCC drug resistance	[101, 114]
	Adoptive immunotherapy	CAR-T/TCR-T cell therapy	Dysregulation of BAs → ①Inhibited CD80/CD86 expression on DCs; ②Induced ROS accumulation; ③Limited the energy supply of TME → CAR-T/TCR-T cell exhaustion → HCC drug resistance	[116, 119, 120]
		TILs/CIK cell therapy	BAs → Promotion of Tregs and M2 macrophages differentiation → Increased the immunosuppressive properties of TME → Inhibition of anti-tumor activity of TILs/CIK cells → HCC drug resistance	[100, 123]

a BCL-2, B-cell lymphoma-2; CAR-T, chimeric antigen receptor T-cell; CD80/CD86, cluster of differentiation 80/86; CIK, cytokine-induced kller cell; CTLA-4, cytotoxic T-lymphocyte antigen-4; CYP3A4, cytochrome P450 3A4; DCs, dendritic cells; EMT, epithelial–mesenchymal transition; GCDC, glycochenodeoxycholic acid; LAG-3, lymphocyte activation gene-3; LCSCs, liver cancer stem cells; MCL-1, myeloid cell leukemia-1; MDR1, multi-drug resistance protein 1; mitoROS, mitochondrial ROS; PD-1, programmed cell death protein-1; PD-L1, programmed death-ligand 1; PXR, pregnane X receptor; TCR-T, T cell receptor-engineered T cell; TGF-β, transforming growth factor-β; TILs, tumor-infiltrating lymphocytes; TIM-3, T-lymphocyte immunoglobulin mucin-3; TME, tumor microenvironment; Tregs, regulatory T cells; UDCA, ursodeoxycholic acid; VEGFR-2, vascular endothelial growth factor receptor-2.

### BAs and sorafenib resistance

Sorafenib is the first targeted therapy approved for the treatment of advanced or unresectable HCC [4]. Sorafenib inhibits receptor tyrosine kinase, such as vascular endothelial growth factor receptor 1–3 (VEGFR 1–3), platelet-derived growth factor receptor-β, and fibroblast growth factor receptor-1, thereby inhibiting tumor angiogenesis and promoting apoptosis [68]. However, HCC patients develop resistance to sorafenib, which reduces its therapeutic efficacy, and the mechanism involves the interaction of several different signaling pathways [69]. Meanwhile, there is a certain correlation between these drug resistance mechanisms and BAs. Li *et al*. [70] and Wu *et al*. [71] found that the increase in BA levels caused by FXR deficiency induces IL-6 expression, which in turn activates the Janus kinase 2-STAT3 signaling pathway, and STAT3 activation positively regulates its downstream targets, the anti-apoptotic proteins B-cell lymphoma-2 (BCL-2) and MCL-1, thus collectively reducing the pro-apoptotic effects of sorafenib. In addition, downregulation of FXR results in elevated expression of β-catenin along with its downstream targets c-Myc and cyclin D1, which promotes tumor cell proliferation and invasion capacity, and this mechanism may exhibit a close association with sorafenib resistance [72, 73]. The binding of PXR to sorafenib activates PXR and promotes the expression of the CYP450 enzymes CYP3A4 and MDR1, which accelerates the clearance or elimination of sorafenib and ultimately leads to sorafenib resistance [74]. Therefore, in HCC, the receptor signaling pathways that affect BA metabolism, such as FXR and PXR, can lead to sorafenib resistance in HCC patients.

### BAs in lenvatinib and regorafenib resistance

Lenvatinib and regorafenib are multi-targeted TKIs that have potent anti-tumor activity against HCC [75, 76]. However, HCC patients can also develop resistance to lenvatinib and regorafenib, with complex and diverse mechanisms, and BAs are also involved in part of the resistance mechanisms [77–79]. The expression of DMEs and transporter proteins is modulated by BA metabolism, which affects the pharmacokinetic properties of lenvatinib, thus reducing its therapeutic efficacy and generating drug resistance. It was found that PXR, a key receptor for BAs, could accelerate drug metabolism by inducing CYP3A4 expression. Of note, CYP3A4 is a key enzyme for lenvatinib metabolism and its expression level is closely correlated with the efficacy of lenvatinb. Therefore, PXR may affect lenvatinb resistance through CYP3A4 [80–82].

In addition, abnormal BA metabolism activates cell survival-related signaling pathways such as phosphoinositide 3-kinase (PI3K)/AKT, which counteracts the pro-apoptotic effect of regorafenib and leads to HCC cells evading drug killing [54, 83]. FXR can promote BA transport from the liver to the bile ducts by inducing the expression of BSEP [84]. If BSEP has functional deficiency, the MDR and MRP gene-encoded transporters will be upregulated to compensate [84]. MDR1 and MRP1 transporters could reduce the intracellular accumulation of chemotherapeutic agents in tumor cells by mediating drug efflux, which can reduce HCC patients' sensitivity to drugs such as regorafenib [85, 86]. Therefore, dysregulation of BA metabolism can also affect the function of BSEP through FXR and upregulate the expression of efflux transporter genes, MDR1 and MRP1, which can cause HCC cells to pump regorafenib out of the cell more quickly, reduce intracellular drug concentration, and cause drug insensitivity.

### BAs and other targeted drugs resistance

Cabozantinib is an oral small molecule multi-target TKI for HCC treatment. A component of BAs, GCDC induces cell stemness in HCC through epithelial–mesenchymal transition (EMT) and activation of the STAT3 signaling pathway [87]. Meanwhile, the study has shown that liver cancer stem cells are resistant to conventional chemotherapeutic drugs and serve as a driving force for tumor recurrence and metastasis [88]. Therefore, BAs could induce resistance to TKI-based targeted chemotherapeutic drugs such as cabozantinib by promoting the maintenance of HCC stem cell-like phenotype. In addition, it is reported that LCA triggers the nuclear factor erythroid 2-related factor 2 pathway, which significantly upregulates hepatic MRP2 and MRP3 expression, thereby enhancing BA efflux [89]. Furthermore, Marin *et al*. highlighted that both MRP2 and MRP3 contributed to the MDR phenotype in HCC. These transporters mediate drug efflux, thereby reducing intracellular drug concentrations, conferring resistance to targeted therapies such as cabozantinib [90].

Ramucirumab and apatinib exert anti-tumor effects by binding to VEGFR2, thereby blocking the migration and proliferation of vascular endothelial cells and suppressing tumor angiogenesis [91–93]. As reported in previous studies, VEGFR-2 expression correlates positively with BA levels. Specifically, metabolic dysregulation-induced aberrant BA accumulation promotes tumor angiogenesis in HCC, which may counteract the therapeutic efficacy of ramucirumab and apatinib [94]. Additionally, the oxidative metabolites of apatinib are predominantly formed in the liver via an NADPH-dependent manner, a process primarily mediated by CYP3A4/5 enzymes [92]. As mentioned earlier, BAs can modulate CYP3A4 expression through PXR, which may consequently impact the therapeutic efficacy of apatinib [39].

## BA metabolism and HCC immunotherapy resistance

Emerging information indicates BAs as pivotal regulators of immunotherapy resistance regulators in HCC. By orchestrating metabolic–immune interplay, BAs establish an immunosuppressive niche that compromises multiple therapeutic modalities. Their pleiotropic effects span from checkpoint molecule dysregulation to adaptive immune cell dysfunction, whereas gut microbiota-mediated BA remodeling further dictates therapeutic vulnerability. These multilayered interferences highlight the necessity of targeting BA signaling to overcome resistance across immune checkpoint inhibitors (ICIs), adoptive therapies, and emerging immunotherapeutic strategies (Table 1).

### ICI resistance

#### Programmed cell death protein-1/programmed death ligand 1 inhibitors

Programmed cell death protein-1 (PD-1) is a commonly used immunosuppressive factor on T cell surface, and its ligand, programmed death-ligand 1 (PD-L1), is over-expressed on tumor cell surfaces, inhibiting the proliferation and actuation of T cells, leading to failure of therapy [95]. Thus blocking the PD-1/PD-L1 pathway could restore immune function, and based on numerous clinical trials, PD-1/PD-L1 checkpoint inhibitors have exhibited remarkable response rates in HCC patients [96]. However, anti-PD-1/PD-L1 monotherapy is ineffective for nearly 80% of HCC patients, so the frequency of response to anti-PD-1/PD-L1 therapies is still unsatisfactory and better knowledge of the tolerance mechanisms of such therapies is urgently needed [97, 98]. At the same time BA metabolism mediates certain related resistance mechanisms.

BA metabolism is intricately linked to the immunosupressive tumor microenvironment (TME). Regulatory T cells (Tregs), a pivotal immunosuppressive subset, suppress anti-tumor immune surveillance and promote immune escape, thereby driving oncogenesis. Furthermore, Tregs diminish the clinical efficacy of PD-1/PD-L1 blockade therapies [99]. Notably, specific BAs may contribute to immunotherapy resistance by modulating Tregs. For instance, isoallo LCA, an LCA derivative, enhances mitochondrial ROS (mitoROS) production, leading to higher FoxP3 levels, a transcription factor vital for Tregs differentiation [100]. This FoxP3-dependent Tregs amplification ultimately attenuates the therapeutic reaction to PD-1/PD-L1 inhibitors in HCC. Furthermore, dysregulated BA metabolism contributes to PD-1/PD-L1 inhibitor resistance by modulating M2 polarization of macrophages. Studies have demonstrated that ursodeoxycholic acid (UDCA) promotes macrophage polarization toward the M2 phenotype [101]. Then M2 macrophages modulate the production and metabolic activity of immunosupressive cytokines, including IL-10 and transforming growth factor-β (TGF-β), thereby counteracting T cell-mediated tumor cytotoxicity [102]. These mechanisms foster an immunosuppressive niche for HCC cells, ultimately conferring resistance to PD-1/PD-L1 blockade therapies.

#### Cytoxic T-lymphocyte antigen-4 inhibitors

Cytotoxic T-lymphocyte antigen-4 (CTLA-4) is also a promising target. CTLA-4, like PD-1, belongs to the CD28 immunoglobulin family, which binds to B7 ligands (CD80, CD86) on antigen presenting cells (APCs); CTLA-4 is more dominant when it competes with CD28 for binding of B7 ligands, which can lead to T-cell inactivation [103, 104]. CTLA-4 inhibitors bind the CTLA-4 molecule with high affinity and block its interaction with the B7 ligand, thereby enabling T cells to recognize and assault tumor cells more effectively [103]. However, HCC patients are equally resistant to treatment with CTLA-4 inhibitors and the associated BAs are involved in modulating some of the resistance mechanisms. The conjugated BA, TCDCA, inhibits mitochondrial respiration, leading to increased ROS and induced T-cell death, which diminishes the CTLA-4 inhibitor-dependent T-cell activation effect and causes therapeutic resistance [66]. In addition, LCA and UDCA could activate endoplasmic reticulum stress and T cell depletion pathways, which further accelerate HCC resistance to CTLA-4 inhibitors [66].

#### Other immune checkpoint inhibitors

T cell activation requires two essential signals: (i) the antigenic signal, mediated by the binding of the T cell receptor (TCR) to the antigen-major histocompatibility complex (MHC) [105]; (ii) the co-stimulatory signal, initiated by the interaction between CD28 on T cells and CD80/CD86 expressed on APCs, particularly dendritic cells (DCs) [106]. Lymphocyte activation gene-3 (LAG-3, also known as CD223), commonly co-expressed with PD-1 on exhausted T cells, suppresses T cell activation by transmitting inhibitory signals rather than directly interfering with antigen recognition. This mechanism promotes immune tolerance and contributes to resistance against ICI activity by blocking the interaction between LAG-3 and its ligands (e.g. MHC class II molecules or fibringen-like protein 1), thereby alleviating T cell suppression [107–109]. Moreover, SBAs such as DCA may impair DC function, potentially reducing CD80/CD86 expression and compromising co-stimulatory signaling. The absence of co-stimulation could diminish T cell responsiveness to LAG-3 inhibitors, even when inhibitory checkpoints are pharmacologically blocked [110–112].

T-cell immunoglobulin and mucin domain-containing protein 3 (TIM-3) promotes tumor progression by inducing M2 polarization of macrophages via exosome-mediated mechanisms, thereby triggering the secretion of immunosuppressive factor [113]. Consequently, blocking TIM-3 signaling impedes M2 polarization, reduces the manufacture of pro-tumorigenic cytokines such as IL-6, and alleviates the immunosuppressive characteristics of the TME. Furthermore, TIM-3 inhibitors suppress IL-6/STAT3 signaling pathway activation in HCC cells, effectively inhibiting tumor proliferation and metastasis, which highlights their therapeutic potential against HCC [114]. As previously mentioned, certain BAs (e.g. UDCA) promote M2 polarization, opposing the pharmacological effects of TIM-3 inhibitors. This functional antagonism suggests a potential link between UDCA and drug resistance to TIM-3-targeted therapy [101].

### Adoptive immunotherapy

Chimeric antigen receptor-T (CAR-T) cell therapy involves genetically engineering a patient's own T cells to express synthetic receptors targeting tumor-specific antigens, enabling precise recognition and elimination of malignant cells [104]. Similarly, TCR-engineered T cells (TCR-T) are modified to recognize tumor-associated antigenic peptides presented by MHC molecules [115]. Despite their therapeutic potential, both CAR-T and TCR-T face significant challenges when treating HCC, whose immunosuppressive TME is characterized by infiltrating Tregs, tumor-associated macrophages (TAMs), and myeloid-derived suppressor cells (MDSCs). These cells secrete inhibitory molecules (e.g. IL-10 and TGF-β) and up-regulate immune checkpoints (e.g. PD-1/PD-L1), collectively impairing CAR-T/TCR-T cell activity and driving therapeutic resistance [116, 117]. BAs further exacerbate this resistance through multiple mechanisms: (i) impaired co-stimulation: BAs suppress CD80/CD86 expression on DCs, limiting co-stimulatory signals essential for CAR-T cell expansion and functionality, ultimately promoting T cell exhaustion [118]; (ii) mitochondrial dysfunction in CAR-T cells: SBAs (e.g. DCA) induce mitochondrial membrane depolarization and ROS accumulation, accelerating CAR-T cell exhaustion and diminishing cytotoxic capacity [66]; and (iii) metabolic competition: the activation, proliferation, and effector functions of CAR-T/TCR-T cells demand substantial energy expenditure. However, BA-driven metabolic disturbances in HCC exacerbate TME nutrient deprivation, hypoxia, and lactate accumulation, thereby restricting energy supply and compromising T cell proliferation and cytotoxicity [116, 119, 120].

In addition, tumor-infiltrating lymphocytes (TILs), a type of leukocyte found in tumors, have the ability to naturally recognize tumor antigens. TIL cell therapy enhances the anti-tumor effect of TILs by expanding them *ex vivo*, which can eliminate residual HCC cells and prevent recurrence after reinfusion [121]. Cytokine-induced killer (CIK) cells are polyclonal immune effector cells with mixed T/NK phenotypes, capable of MHC-unrestricted tumor cytotoxicity [122]. CIK cell therapy, which involves *ex vivo* expansion of peripheral blood monouclear cells, has the potential to prolong recurrence-free survival and overall survival when used as adjuvant therapy after resection [122]. The anti-tumor activity of TILs or CIK cell therapy is suppressed by immunosuppressive components in TME of HCC, such as Tregs and M2-polarized macrophages, leading to therapy resistance [123]. Although no studies have directly investigated the impact of BAs on TIL/CIK therapy efficacy, evidence previously discussed indicates that specific BAs promote the differentiation of Tregs and M2 macrophages [100]. This BA-driven enhancement of immunosuppressive cell populations may theoretically exacerbate TME-mediated inhibition of TILs and CIK cells, thereby further contributing to HCC resistance against these cellular therapies.

### Novel immunotherapies for HCC

Tumor vaccines aim to elicit tumor-specific immunity by delivering antigenic targets (neoantigens/tumor-associated antigens) to APCs, thereby activating CTLs. Despite advances in mRNA and personalized neoantigen platforms, therapeutic efficacy remains limited by immunosuppressive TME and inadequate immune activation [124]. Despite the lack of direct evidence linking BAs to tumor vaccines efficacy, emerging evidence suggests their potential role in modulating anti-tumor immunity through multifaceted mechanisms: BAs activate FXR and TGR5 on DCs, altering antigen-presenting capacity and cytokine secretion profiles (e.g. reduced IL-12 and elevated IL-10) [125]. SBAs, such as DCA, exacerbate DC dysfunction by inducing mitoROS overproduction, thereby hindering DC maturation [66]. Concurrently, BA-FXR signaling promotes Tregs differentiation while suppressing CTL activity via upregulation of immune checkpoints (PD-1/CTLA-4) in HCC murine models [126, 127]. Notably, pharmacological sequestration of BAs enhances CD8+ T cell infiltration in vaccine-treated tumors, highlighting the therapeutic potential of BA modulation [128, 129]. The gut microbiota further amplifies this interplay, as BA-metabolizing bacteria dynamically shape systemic BA pools to influence vaccine-primed T cell responses [130]. Preclinical studies show that fecal microbiota transplantation from high-BA donors reduces tumor vaccine efficacy, underscoring the gut–liver axis as a critical determinant of immunotherapy outcomes [131]. These findings position BA metabolism as a dual target for enhancing tumor vaccine efficacy through combinatorial strategies targeting FXR/TGR5 signaling and microbiota modulation.

## Therapeutic strategies targeting BA metabolism

### Therapeutic potential of targeting BA metabolic pathways

In HCC, BA receptor agonists exert multifaceted therapeutic effects by modulating inflammatory, fibrogenic, and oncogenic pathways. However, most of them are still in the transition stage from preclinical to early clinical trials. The FXR agonist obeticholic acid (OCA) induces cell cycle arrest and suppresses tumor cell invasion via STAT3 dephosphorylation and SOCS3 upregulation, concomitant with reduced IL-6/STAT3 signaling and pro-inflammatory cytokine (IL-1β, IL-6) secretion [132]. Currently, multiple clinical trials using OCA are being conducted to assess its safety and efficacy in HCC treatment. In a Phase III clinical trial, patients with NASH-induced compensated cirrhosis were randomly assigned to receive daily oral administration of 10 or 25 mg of OCA tablets or placebo for 18 months. The study found that OCA improved liver fibrosis by at least one stage and did not exacerbate NASH. This provides important data supporting the potential application of OCA in HCC treatment [133]. With the exception of OCA, most other FXR agonists with HCC therapeutic effects are still in preclinical research, including GW4064, Px20606, and Int-767[134]. In particular, GW4064 delays HCC progression by activating SOCS3 to block STAT3-driven oncogenesis [54]. The application of GW4064 in HCC treatment is still in the research stage. Preclinical studies showed that GW4064 has significant therapeutic effects on HCC in various animal models and exhibits anti-tumor activity *in vitro*, inhibiting the proliferation, migration, and invasion of HCC cells. Therefore, GW4064 has potential application value in the treatment of HCC, and further clinical trials are needed to validate its efficacy [134, 135].

TGR5 agonist (e.g. INT-777) mitigates NF-κB-mediated inflammation and oxidative stress in macrophages, thereby attenuating chronic inflammation-driven hepatocarcinogenesis [136, 137]. PXR activation (e.g. rifampicin) enhances detoxification by recruiting hepatocyte nuclear factor 4α/steroid receptor coactivator-1 (HNF4α/SRC-1) to the CYP3A4 promoter while competitively inhibiting FXR-SHP signaling, a dual mechanism that reduces BA toxicity [138]. These drugs with potential for HCC treatment are still in the preclinical exploration stage. In the future, preclinical HCC model validation will need to be conducted to further advance clinical translation [139, 140].

Gut microbiota modulation synergizes with BA-targeted therapies. Lipopolysaccharide (LPS) activates hepatic Kupffer cells via Toll-like receptor 4 (TLR4), triggering tumor necrosis factor-α (TNF-α)/IL-18 overproduction and perpetuating inflammatory liver injury [141]. Probiotics (e.g. Lactobacillus, Bifidobacterium) counteract this cascade by restoring microbial balance, enhancing intestinal barrier integrity, and suppressing LPS-TLR4 signaling, thereby attenuating steatosis, inflammation, and fibrosis [142]. Notably, carcinogenic SBAs (e.g. DCA, LCA) promote HCC by disrupting epithelial barriers and inducing DNA damage [48]. However, beneficial microbiota inhibits 7α-dehydroxylation of PBAs, reducing fecal DCA levels and associated genotoxicity [143]. Prebiotics, such as inulin and oligofructose, selectively nourish commensal Bifidobacterium populations, fostering an anti-inflammatory milieu through immunomodulatory metabolites (e.g. short-chain fatty acids). Preclinical models demonstrate that inulin supplementation reduces hepatic inflammatory markers and rebalances pro-/anti-inflammatory cytokine networks, effectively decelerating HCC development [144]. By targeting gut–liver axis dysregulation, these interventions disrupt chronic inflammation, a pivotal driver of hepatocarcinogenesis. For a comprehensive overview of the mechanisms by which pharmacological modulation of BA metabolism enhances HCC therapy, refer to Table 2.

**Table 2.** Mechanisms of targeting BA metabolism drugs to improve HCC treatment^a^.

Drug type	Specific drugs	Effects	Ref.
FXR agonists	OCA	①Upregulation of STAT3 dephosphorylation and SOCS3; ②Reduction of IL-6/STAT3 signaling → Induction of cell cycle arrest and inhibition of tumor cell invasion → Inhibition of HCC cell growth	[132]
	GW4064	Regulation of SOCS3 → Blocking the STAT3 pathway → Delaying the progression of HCC	[54]
TGR5 agonists	INT-777	Inhibition of NF-κB → Reduced inflammatory response and oxidative stress → Delaying the progression of HCC	[136, 137]
PXR agonists	Rifampicin	Recruitment of HNF4α/SRC-1 to CYP3A4 promoter → ①Enhancement of detoxification; ②Competitive suppression of FXR-SHP signaling → Reduction of BAs toxicity → Delaying the progression of HCC	[138]
Probiotics	Lactobacillus/Bifidobacterium	Reduced dehydroxylation of PBAs → Reduction of DCA concentration → Enhanced intestinal barrier function → Reduced expression of LPS and TLR4 → Delaying the progression of HCC	[141, 142]
Prebiotics	Inulin/oligofructose	Nourishing symbiotic bifidobacterial populations → Generation of immunomodulatory metabolites → Suppression of liver inflammation → Delaying the progression of HCC	[144]

a HNF4α, hepatocyte nuclear factor 4α; LPS, lipopolysaccharide; SOCS3, suppressor of cytokine signaling 3; SRC-1, steroid receptor coactivator-1; SHP, small heterodimer partner; TLR4: Toll-like receptor 4.

### Combined treatment strategies

While targeting BA metabolism holds promise in HCC treatment, monotherapy limitations, such as FXR nuclear translocation attenuation and acquired resistance, highlight the necessity for combination regimens [134]. Preclinical studies demonstrate that UDCA synergizes with sorafenib by suppressing STAT3 phosphorylation to downregulate cyclin-dependent kinase (CDK) anti-apoptotic networks, and activating ROS-ERK signaling to amplify pro-apoptotic BCL-2 suppression [145]. Similarly, GW4064 enhances oxaliplatin sensitivity by restoring SHP expression and triggering BCL-2-associated X protein/caspase-3-mediated apoptosis, while OCA co-administration with cisplatin upregulates the FXR target transcription elongation factor A2, selectively protecting hepatocytes from cytotoxicity [134, 146, 147]. Immunologically, UDCA degrades TGF-β to inhibit Tregs differentiation and synergizes with anti-PD-1 by augmenting tumor-specific CD8+ T cell memory, correlating with improved objective response rates in retrospective cohorts [148]. Collectively, these findings position BA pathway modulation as a linchpin for overcoming therapeutic resistance through mechanistically rational combinations.

## Discussion and future prospects

BA metabolism dysregulation has appeared as a pivotal driver of HCC pathogenesis and treatment resistance. BAs directly modulate HCC cell proliferation, apoptosis, and metabolic reprogramming through NRs (FXR and PXR) and membrane receptors (TGR5), whereas gut microbiota-derived SBAs (e.g. DCA, LCA) indirectly induce DNA damage, inflammatory cascades, and pro-oncogenic signaling. FXR deficiency increases BA levels, which activate pro-inflammatory pathways (IL-1β/NF-κB) to improve HCC invasion. Concurrently, HFD-induced gut dysbiosis disrupts intestinal barrier integrity, leading to the elevated level of its metabolite TCA, which further induces hepatic inflammatory response and cell proliferation, accelerating the HCC development. In targeted therapy resistance, BAs counteract sorafenib/lenvatinib-induced apoptosis via STAT3 and PI3K/AKT survival signaling, while upregulating DMEs (CYP3A4) and efflux transporters (MDR1) to reduce intracellular drug concentrations. Immunologically, BA imbalance promotes Tregs differentiation and M2 macrophage polarization, fostering immune evasion. For instance, LCA derivatives amplify Tregs immunosuppression via mitoROS–FoxP3 axis activation, whereas UDCA paradoxically improves M2 polarization through TLR4/NF-κB in specific contexts, thereby collectively undermining PD-1 inhibitors and adoptive immunotherapies.

Targeting BA metabolism demonstrates a transformative avenue to combat therapeutic resistance in HCC. Pharmacological modulation of BA receptors, such as FXR agonists (e.g. OCA) and TGR5 agonists (e.g. INT-777), restores BA homeostasis, which exerts anti-fibrotic and tumor-suppressive effects through receptor-specific signaling. Concurrently, interventions that target the gut–liver axis, including probiotics and prebiotics, attenuate HCC progression by dampening pro-inflammatory cytokine networks (e.g. TNF-α) and mitigating DCA-induced genotoxicity. Emerging preclinical evidence further emphasizes the potential of rational combination therapies: UDCA synergizes with sorafenib to amplify ERK/STAT3-mediated apoptosis, whereas its co-administration with anti-PD-1 reinvigorates anti-tumor immunity by suppressing Tregs activity.

In conclusion, dysregulated BA metabolism is intricately associated with HCC pathogenesis and therapeutic resistance. As the third leading cause of cancer-related mortality globally, HCC remains a formidable clinical challenge. Despite advancements in targeted treatments and immunotherapies, pervasive drug resistance has markedly limited their efficacy. Considering the central role of BA metabolism in hepatic pathophysiology, future research should prioritize the following interconnected frontiers. First, identifying subtype-specific mechanisms by which individual BA species drive distinct resistance phenotypes, particularly how dynamic fluctuations in BA profiles temporally modulate therapeutic susceptibility. Second, developing rational combination strategies that integrate BA pathway modulation with existing therapies, which is a vastly underexplored yet therapeutically promising approach. Finally, investigating the effect of BA remodeling on emerging immunotherapies, including oncolytic viruses and neoantigen vaccines, to counteract microenvironment-induced immune evasion. Transformative strategies may emerge to surmount treatment barriers in HCC management by unraveling these BA-centric resistance networks and fostering innovative therapeutic synergies.
